# Delayed diagnosed trauma in severely injured patients despite guidelines-oriented emergency room treatment: there is still a risk

**DOI:** 10.1007/s00068-021-01754-5

**Published:** 2021-07-29

**Authors:** Arnold J. Suda, Kristine Baran, Suna Brunnemer, Manuela Köck, Udo Obertacke, David Eschmann

**Affiliations:** 1 Department of Orthopaedics and Trauma Surgery, AUVA Trauma Center Salzburg, Academic Teaching Hospital of Paracelsus Medical University, Dr. Franz-Rehrl-Platz 5, 5010 Salzburg, Austria; 2 Department of Orthopaedics and Trauma, Medical Faculty, University Medical Center Mannheim, Mannheim of Heidelberg University, Theodor-Kutzer-Ufer 1-3, 68167 Mannheim, Germany

**Keywords:** Missed injury, Delayed diagnosed injury, Trauma algorithm, ATLS^®^, Number needed to fail

## Abstract

**Purpose:**

Emergency trauma room treatment follows established algorithms such as ATLS®. Nevertheless, there are injuries that are not immediately recognized here. The aim of this study was to evaluate the residual risk for manifesting life-threatening injuries despite strict adherence to trauma room guidelines, which is different to missed injuries that describe recognizable injuries.

**Methods:**

In a retrospective study, we included 2694 consecutive patients admitted to the emergency trauma room of one single level I trauma center between 2016 and 2019. In accordance with the trauma room algorithm, primary and secondary survey, trauma whole-body CT scan, eFAST, and tertiary survey were performed. Patients who needed emergency surgery during their hospital stay for additional injury found after guidelines-oriented emergency trauma room treatment were analyzed.

**Results:**

In seven patients (0.26%; mean age 50.4 years, range 18–90; mean ISS 39.7, range 34–50), a life-threatening injury occurred in the further course: one epidural bleeding (13 h after tertiary survey) and six abdominal hollow organ injuries (range 5.5 h–4 days after tertiary survey). Two patients (0.07% overall) with abdominal injury died. The “number needed to fail” was 385 (95%–CI 0.0010–0.0053).

**Conclusion:**

Our study reveals a remaining risk for delayed diagnosis of potentially lethal injuries despite accurate emergency trauma room algorithms. In other words, there were missed injuries that could have been identified using this algorithm but were missed due to other reasons. Continuous clinical and instrument-based examinations should, therefore, not be neglected after completion of the tertiary survey.

**Level of evidence:**

Level II: Development of diagnostic criteria on the basis of consecutive patients (with universally applied reference “gold” standard).

## Introduction

Emergency trauma room diagnostics include structured clinical patient assessment according to guidelines provided by Advanced Trauma Life Support (ATLS^®^) with mandatory procedures (blood cell count, extended Focused Assessment Sonography for Trauma (eFAST)) and potential adjuncts (X-ray, whole-body computer tomography) [1]. Some injuries need time to develop and might not be detected in the primary assessment, whereas other injuries may not be diagnosed due to other manifest injuries.

Taking this into account, the emergency trauma room physician should keep some parameters in mind for decision-making: trauma mechanism, specific lead symptoms for injuries, time to emergency room, hospital resources, sensitivity and specificity of diagnostic tools, prioritization of diagnostic steps, and delegation of responsibilities in rapid patient assessment.

Structured trauma training or certifications may help here to “treat first what kills first”, “do no further harm”, and “re-assess” [2]. Whole-body CT scan can be used to identify brain injuries, but there are insufficiencies in abdominal injury diagnostics [3, 4]. Extended fast assessment sonography for trauma (eFAST) can identify thoracic and abdominal pathologies [5, 6].

In 2018, data from the German Trauma Registry revealed that in 79.5% of cases, a whole-body CT scan was performed on severely injured patients [7]. There are no mandatory requirements, but widely accepted emergency room treatment procedures include time management, emergency room equipment (including sonography and CT scan), personnel training standards, and a structured survey. In 2009, Huber-Wagner et al. showed the life-saving impact of whole-body CT scan and determined that the “number needed to scan” to identify one more survivor was 17 [8]. Early total care and damage control surgery/orthopedics were influenced by this publication [9]. Missed injury is most commonly defined as an injury missed at initial assessment up to 24 h, including primary and secondary surveys and emergency intervention [10].

Hajibandeh et al. recommend differentiating between the terms *“missed injury detection rate” and “missed injury rate”*. [11] Keijzers et al. defined “Delayed Diagnosed Injuries” (DDI) type I for DDI found during the tertiary survey, type II for DDI found during admission, but after tertiary survey and type III for “DDI found after discharge” [10]. Ferree et al. recommend similar wording and Tammeling et al. extend this with type 0 (diagnosis before tertiary survey) [12, 13].

The National trauma database of the American College of Surgeons defines “missed injury” as an injury-related diagnosis discovered after the initial workup is completed and the admission diagnosis is determined [2]. Risk factors for missed injuries are higher Injury Severity Score ISS [14], altered mental status, intubation, need for intensive care, and emergency surgery [12, 15–17]. Therefore, X-rays must be carefully reviewed, any examination that is not clear must be repeated, and serial examinations should be continued for the entire clinical course. Delayed injuries can be found in peripheral extremity regions such as the feet and hands [12, 18, 19] but also in the abdomen and cranium [11, 13].

According to Pfeifer and Pape (2008) primary missed injuries occur in a mean of 10% (1.3–39%) of presenting cases [20]. Tertiary survey reduces the incidence of missed injuries and can be used as an indicator for diagnostic quality. However, there is no clear timepoint for this examination [2, 11–13, 16, 21, 22].

Schneck et al. showed a delayed diagnosis of abdominal injuries of between 0.9% and 5%, mostly without any therapeutic consequences [23, 24].

ATLS^®^ guidelines are widely accepted. Over-triage is known to be superior, and performing a tertiary survey is mandatory to identify intracranial or intra-abdominal bleeding [1].

Repeated brain CT scan in intubated patients and repeated abdominal sonography in all patients can be recommended as part of further clinical examination, but an exact point of time for this cannot be given. Six hours after admission is widely used but is not evidence-based.

Injuries that occur after a second CT scan or sonography are in the group of remaining risk.

The aim of this study was to identify the number needed to fail (NNF): what is the risk rate of suffering from a life-threatening body cavity injury despite having a negative structured trauma-room treatment, including whole-body CT scan and tertiary survey? How many treatments are necessary to manifest this risk in one single patient?

## Patients and methods

In this retrospective study, we included all patients who were admitted to the emergency trauma room of a level-I university trauma center over a 4-year period (January 2016 and December 2019). All patients received inpatient treatment with clinical assessment according to ATLS^®^ (primary, secondary, tertiary survey), blood cell count, eFAST, repeat eFAST and, dependent on the injury pattern, radiological examination or whole-body CT scan for trauma except for patients who were pregnant, young children or suffered from obvious single-site injury. Data sets from the electronic hospital information system (SAP), including radiological data of patients who received inpatient treatment, were evaluated. We excluded patients with insufficient data sets or outpatient treatment. Patients who needed emergency surgery during their hospital stay for additional injury found during emergency trauma room treatment were identified and analyzed. The remaining risk for life-threatening injuries and numbers needed to treat, fail or harm were calculated.

Statistical analysis with demographic data analysis, Student´s *t* test and Chi-square test was performed using SPSS (Version 24.0), a *p* value < 0.05 was defined as statistically significant.

The regional and institutional ethical committee approved the study (MA-2020-801R).

## Results

3124 patients (1034 females, 2090 males, 67%) were admitted to the emergency trauma room at University Medical Center Mannheim, Germany, between January 2016 and December 2019 (mean age 44 years, range 0–99). 2694 patients received inpatient treatment with clinical assessment according to ATLS^®^ (primary, secondary, tertiary survey), whole-body CT scan for trauma (except 367 patients = 13% who were pregnant, young children or suffered from obvious single-site injury), eFAST and repeat eFAST. 430 patients received outpatient treatment for minor injuries and were not re-admitted to hospital, and 125 of them (29%) did not receive a whole-body CT scan. Reasons were: obvious isolated injury (brain injury), clinical absence of trauma symptoms in pregnant females, adolescents or children after low energy trauma, inpatient treatment with re-assessment, immediate emergency surgery, and death in the emergency room.

176 patients (6.6%) died in the period up to 8 h after admission. 110 patients (4.08%) showed an intra-abdominal injury (AIS > 2), 7 of them died in the period up to 48 h after admission due to severe vessel injury. Of these 110 patients, 102 (92.7%) experienced blunt trauma, 66.3% in motor vehicle accidents. Overall, 17 patients suffered from bowel or mesentery injuries due to blunt abdominal trauma (incidence = 0.63%). We identified 83 injuries of parenchymatic organs (spleen, liver, kidney, and pancreas), 15 intra-abdominal vessel injuries, and 18 hollow organ (bowel, stomach, and bladder) and mesentery injuries. Two small bowel injuries occurred in penetrating trauma. We found severe brain trauma (GCS < 12, emergency surgery, AIS > 2) in 368 patients (13.66%), 111 of them (30.16%) died within 48 h after admission.

Seven patients (0.26%; mean age 50.4 years, range 18–90, 2 females, 5 males; mean ISS 33.7, range 17–50) underwent delayed emergency surgery due to an injury that was not identified despite a correct emergency trauma room algorithm including imaging and admission to ICU. Six of these patients showed intra-abdominal injury, one an epidural bleeding (Table 1). Patients 1 and 6 showed hemodynamic instability within the first two hours after finishing the tertiary survey, which led to intubation. In a repeat eFAST, new intra-abdominal free fluid could be identified and explorative laparotomy was performed. Patient 6 (90 years) did not survive. Neither patient could be classified according to Kejzers’ DDI [10].

**Table 1:** Data of the seven patients with delayed emergency surgery

Patient Nr	Sex, age	Trauma mechanism	Initial ISS	GCS	Injury	Diagnostic method	Time between tertiary survey and surgery	Admission after ER	Initial resucitation	Result
1	M, 49	Fall > 3 m	17	10	Femur fracture, pneumothorax, brain injury, bowel contusion, bleeding	Sonography	4.5 h	ICU	Fluids (responder)	Discharged day 10
2	F, 51	MVA	17	14	Hemato-pneumothorax, pelvic fracture, bowel perforation	Ascites punction	1 day	ICU	Fluids (responder)	Discharged day 20
3	F, 73	MVA	48	3	Brain injury, multiple fractures, thoracic trauma, spine injury, bowel perforation with peritonitis	CT scan	8 days	ICU	Fluids (responder) Blood (ICU)	Death day 18
4	M, 30	MVA	43	3	Brain injury, multiple fractures, thoracic injury, spine injury, bowel perforation	Sonography Ascites punction, CT scan	2 days	ICU	Fluids (responder) Blood (ICU)	Discharged day 38
5	M, 37	MVA	34	3	Multiple fractures, spleen injury, pneumothorax, bowel perforation with peritonitis	Ascites punction	6 days	ICU	Fluids (responder) Blood (ICU)	Discharged day 19
6	M, 90	MVA	50	10	Brain injury, hemato-pneumothorax, multiple fractures, spine injury, bowel perforation	Sonography	5.5 h	ICU	Fluids (responder) Blood (ICU)	Death day 11
7	M, 18	MVA	27	14	Pneumothorax, femur fracture, epidural bleeding	CT scan	13 h	ICU	Fluids (responder)	Discharged day 8

In patient 2 (awake, DDI type I), abdominal pain occurred after 24 h and further diagnostics and therapy could be initiated. In patient 7 (awake, DDI type I), nausea and vomiting led to a repeat CT scan of the brain 13 h after admission and intra-cerebral bleeding could be identified and treated surgically. In patients 3, 4, and 5 (DDI type II), clinical manifestation of perforated hollow organ was delayed significantly: Patient 3 showed rising signs of infection in the blood cell count but without clinical signs of acute abdomen. After 3 days, a repeat CT scan of the abdomen was performed, and free intra-abdominal fluid was diagnosed with known presence of pelvic fracture and hematoma. Another CT scan after 8 days showed contrast fluid leakage, and explorative laparotomy was performed but at this time with signs of severe sepsis. The patient died. Patient 4 (awake, traumatic spinal cord injury with complete thoracal paraplegia) showed signs of acute abdomen on day 2 and free intra-abdominal fluid in a repeat eFAST. In re-CT of the abdomen, bowel perforation could be seen and treated. Patient 5 (awake, spleen injury, conservative treatment) did not show clinical remission over 6 days. A repeat CT scan showed free intra-abdominal fluid. Both patients who died had a high injury severity score (ISS 48 and 50) and higher age (73 and 90). The remaining risk for patients in our cohort was 0.26% (95%–CI 0.0010–0.0053), the “number needed to fail” was 385.

## Discussion

The acute diagnostic safety of hollow organ injuries in emergency trauma room according to guidelines is high: the structured trauma assessment only failed to identify life-threatening injury in 7 of 2694 patients. Two of the 7 patients died. All cases of abdominal injuries showed bowel perforation. All 7 patients were treated in hospital. Missed or delayed diagnosed injuries are known, but to our knowledge, this is the first study to quantify the remaining risk of life-threatening injuries, which is different from missed or delayed diagnosed injuries.

Under- and over-triage or over-diagnostics with the inclusion of too many patients in an emergency trauma room algorithm has recently been discussed. However, no changes of algorithms or guidelines have been implemented so far—under-triage of 5% and over-triage of 25–35% are stated as acceptable, and more than 25% of “too many” alarms should be tolerated [25]. Huber-Wagner et al. mentioned a number needed to treat of 17 for whole-body CT scan: 17 patients need to be investigated to save one patient’s life [8]. German trauma registry data shows 11.4% overall lethality [7]. Only a retrospective study design in a large cohort treated with exactly the same algorithm can identify any remaining risks. In a prospective setting, the presence of any injury could be suspected in a tertiary survey, and a bias would be created. Although whole-body trauma CT scan is widely used, clinical examination according to a guideline-related algorithm is crucial. Overall, hollow organ injuries in blunt abdominal trauma are relatively rare, with rates of between 2.9% and 5% [26–28]. Lawson et al. analyzed 26,264 trauma patients and found 90 patients (0.34%) with delayed diagnosis [29]. Most missed injuries were bowel and mesentery (16, 17%: mostly present on day 2 after admission). Small bowel injuries show slow and occult presentation with peritonitis leading to sepsis in some cases: other patients survive [30].

Especially in blunt abdominal trauma, bowel injuries lead to increased morbidity and mortality. The risk of “missed injury” is higher in blunt compared to penetrating abdominal trauma [31–33]. In 2000, Fakhry et al. reported 198 patients with 9 dead due to delayed diagnosed small bowel perforations (SMP) [28]. In 2019, the same authors presented a study of 127,919 trauma patients with 77 suffering from SBP (incidence 0.06%, 8.43 h to surgical intervention). One patient died, the mortality rate was 1.4%, and longer time to surgery was correlated to mortality [34]. Clinical assessment without whole-body CT trauma scan led to false-negative laparotomies and diagnostic peritoneal lavage of up to 40% in the past: several authors concluded that laparotomy is not mandatory in patients with intra-abdominal free fluid in CT scan and blunt abdominal trauma without injury of parenchymatous organs [26, 35, 36]. In these cases, clinical re-assessment and a repeat CT scan is recommended [37]. Scaglione and others recommended clinical observation, monitoring, surgical expertise, and CT scan for the decision-making process in patients with blunt abdominal trauma [37–39]. Yoong et al. found high sensitivity of whole-body CT scan with a missed injury rate of 2.4% [40].

In our study, the residual risk appears between several hours and 8 days after hospital admission. Intubation or reduced vigilance were not risk factors, and high ISS was associated with higher mortality. Re-evaluation is the key to catching all injuries. We could not identify any suggestions for improvement regarding therapy or diagnostics. The low mortality rate is related to central Europe, modern technical equipment, low mass casualty rates, and low gunshot/stab wound injury rates.

We recommend classifying missed injuries in

Delayed diagnosed injuries DDI

A = after trauma room treatment

B = during tertiary survey

C = after tertiary survey until discharge from hospital

With subgroups

1. potentially life-threatening
2. therapeutically relevant injuries
3. facultative relevant injuries

All patients with manifest risk in our study were only classified as B1 and C1.

We cannot recommend a certain point of time for the tertiary survey based on our data. However, a repeated eFAST or CT scan is not an alternative for a correct clinical re-assessment, as described in the tertiary survey.

In our study, all seven detected patients showed ISS > 16 and initially diagnosed spinal cord injury, spleen rupture, or pelvis fracture might have led to distraction compared to other injuries even there was no sign of injury in initial clinical and instrument-based diagnostics.

## Data Availability

With corresponding author due to ethical committee vote.
